# Supplementary material for: The inflammatory path toward type 1 diabetes begins during pregnancy
Source: Nat Commun. 2026 Jan 7;17:979. doi: 10.1038/s41467-025-67712-6 (PMC12847856; doi:10.1038/s41467-025-67712-6)
Supplement: Supplementary file 1 — Supplementary Information [file 41467_2025_67712_MOESM1_ESM.pdf]

Supplementary Information PDF file

The inflammatory path toward type 1 diabetes begins during pregnancy

First Author: Angelica P. Ahrens

Corresponding: Eric W. Triplett, and Johnny Ludvigsson

a

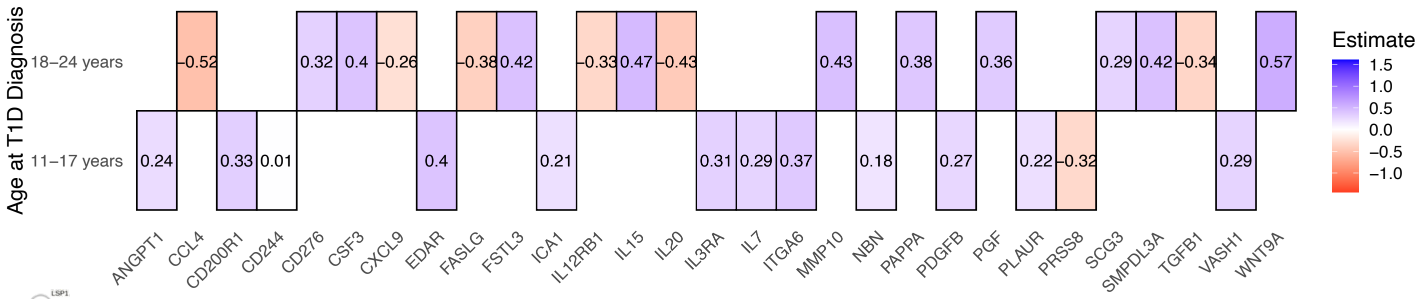

b

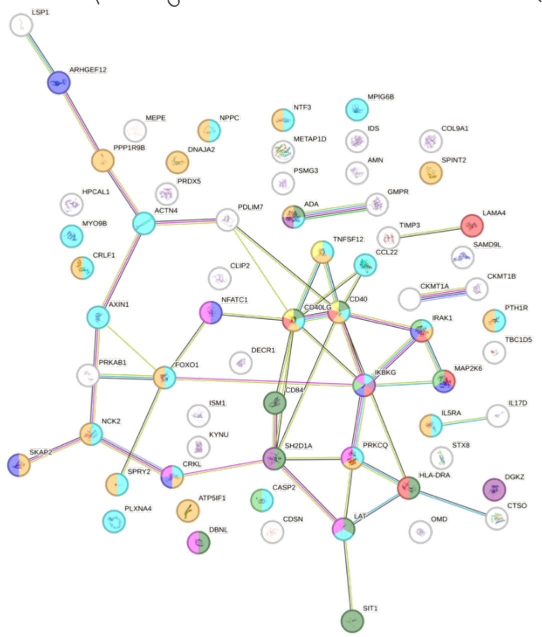

Biological Process (Gene Ontology)

GO-term	description	count in network	strength	signal	false discovery rate
GO:0002250	Adaptive immune response	9 of 359	0.88	0.61	0.0038
GO:0007166	Cell surface receptor signaling pathway	24 of 2040	0.55	0.59	0.00022
GO:0050856	Regulation of T cell receptor signaling pathway	4 of 45	1.43	0.58	0.0137
GO:0042127	Regulation of cell population proliferation	20 of 1669	0.56	0.56	0.00067

KEGG Pathways

pathway	description	count in network	strength	signal	false discovery rate
hsa05145	Toxoplasmosis	7 of 103	1.31	1.25	2.45e-05
hsa05135	Yersinia infection	7 of 124	1.23	1.16	4.06e-05

Reactome Pathways

pathway	description	count in network	strength	signal	false discovery rate
HSA-168638	NOD1/2 Signaling Pathway	4 of 35	1.54	0.67	0.0076
HSA-5676594	TNF receptor superfamily (TNFSF) members mediating non-canoni...	3 of 17	1.73	0.57	0.0170

WikiPathways

pathway	description	count in network	strength	signal	false discovery rate
WP69	T-cell receptor signaling pathway	6 of 90	1.31	0.93	0.00057

Supplementary Fig. 1 Proteomic differences in future T1D diagnosed from 11-24 years of age and Protein-Protein Interaction Network in Early-Diagnosed T1D. a) Proteomic differences between future cases and controls (n=286). Protein differences specific those diagnosed at 11-17 years (n=60) or 18-24 years (n=29) are indicated. Wilcoxon p-values are indicated, before FDR. b) Protein-protein interactions (curated from databases or shown experimentally) by STRING, with edges showing associations that are deemed specific and meaningful by contribution to a shared function. The proteins found to be significant in early-diagnosed T1D cases (diagnosed with T1D by age five), after FDR correction are prioritized (Supplementary Tables 8-9). Colored nodes represent mapping to Gene Ontology, KEGG, Reactome, or WikiPathway databases, as indicated.

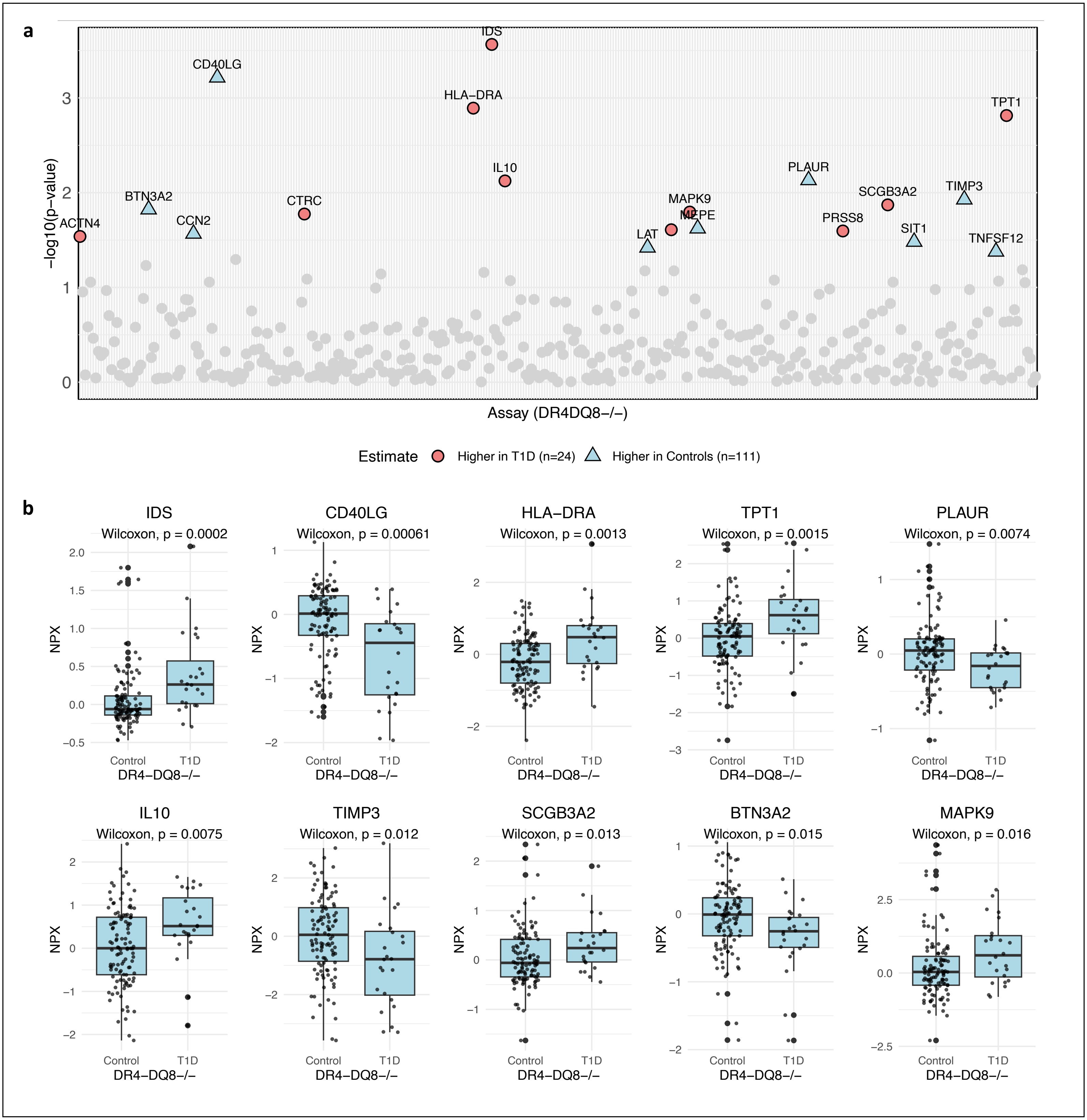

Supplementary Fig. 2. Proteomic differences in T1D and controls, specifically those lacking DR4-DQ8 HLA alleles: a) Significant proteins between cases (n=24) and controls (n=111), both lacking DR4DQ8. None are significant after false discovery rate (FDR) adjustment; b) Significant proteomic markers in T1D and controls lacking the DR4-DQ8 allele (before FDR adjustment). Boxplots show the median (line) and interquartile range (box, 25th–75th percentile) to demonstrate the data distribution. Whiskers extend to the most extreme values

within 1.5 × interquartile range (IQR) of the lower and upper quartiles; points beyond the whiskers are plotted as outliers. Values are displayed as normalized protein expression (NPX) values.

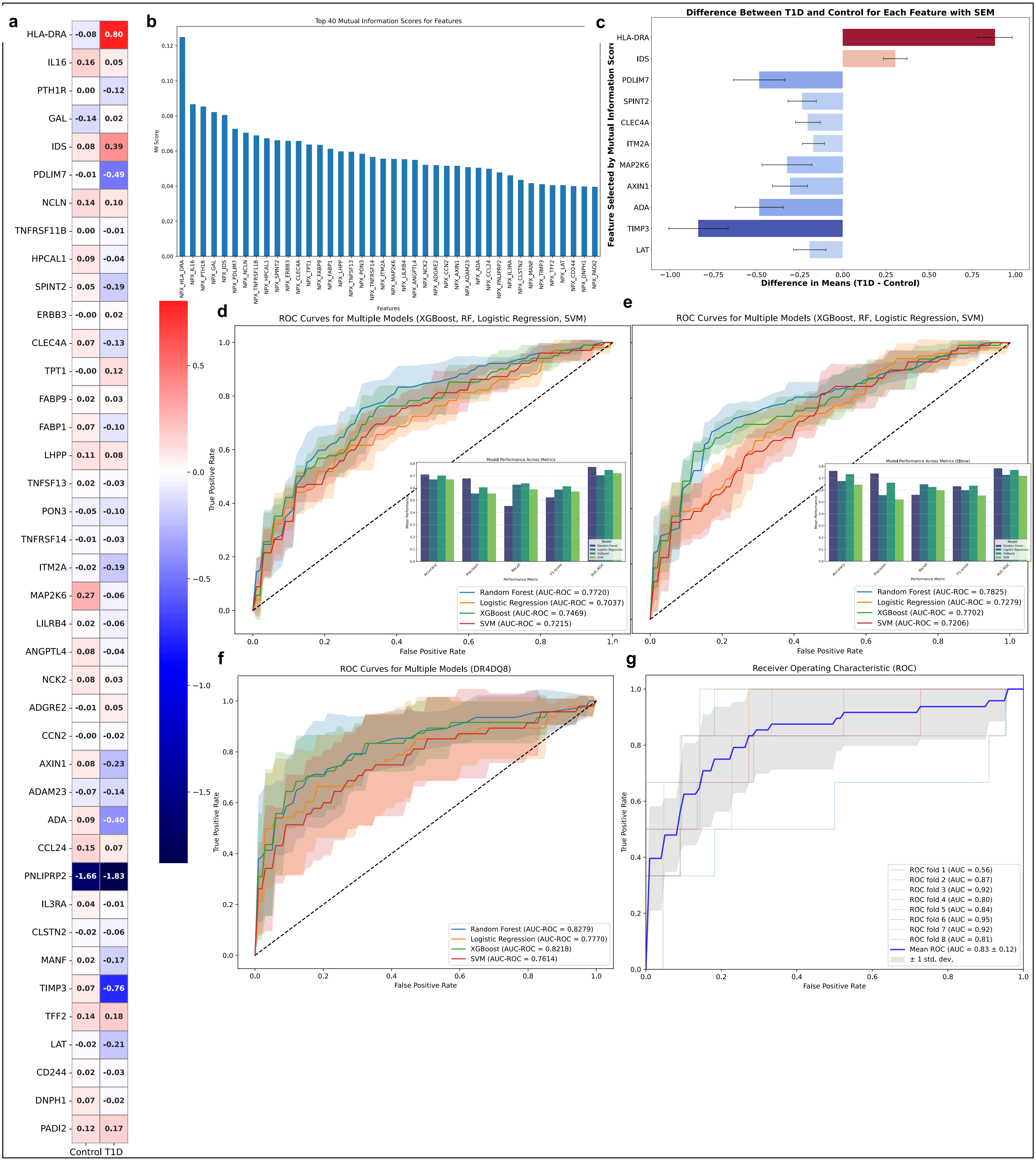

Supplementary Fig. 3. Machine learning (ML) to predict type 1 diabetes (T1D) from selected proteins: a) Mean normalized protein expression (NPX) values for the top proteins contributing to the ML by mutual information (MI) scoring, separated by control and future T1D in the Olink case/control cohort (n=432; cases, n=146; controls,

n=286). b) MI scores for proteins explaining the model. c) Difference in means between groups, with standard error of the mean (SEM). Comparison of ML and traditional model performance, training on 70% of the dataset (n=275) and testing on 30% (n=118), using d) top 40 proteins, and e) top 52 proteins, selected by the elbow method. For XGBoost, the baseline parameters are scale_pos_weight=4.0, random_state=42. Stratified k-fold cross-validation maintains the class distribution (n_splits=5, shuffle=True, random_state=42). ROC curves are averaged across folds using interpolation on fixed set of false positive rates, with mean ROC curves and shaded standard deviation. Accuracy, precision, recall, F1 score, and AUC-ROC are indicated as means. f) ML and traditional model performance, predicting future T1D in cases with human leukocyte antigen (HLA) DR4-DQ8 alleles (n=68) from all controls (irrespective of HLA genotype, n=268), trained with 70% of the dataset and tested with 30%, using class weight multiplier 4.2 (based on 68 T1D / 286 controls). Stratified k-fold cross-validation is again applied, with the top 40 proteins by MI as predictors. g) Hyperparameter tuning using grid search with cross-validation (e.g., scale_pos_weight = 10, max_depth = 3), with XGBoost as the final. Stratified 8-fold cross-validation ensures balanced target distribution with a fixed random seed for reproducibility. Mean ROC curve and 95% confidence region (standard deviation) are shown. Abbreviations: ADA, adenosine deaminase; AUC-ROC, area under the receiver operating characteristic curve; AXIN1, axin-1; CLEC4A, C-type lectin domain family 4 member A; HLA-DRA, major histocompatibility complex, class II, DR alpha; ITM2A, integral membrane protein 2A; LAT, linker for activation of T cells; MAP2K6, mitogen-activated protein kinase kinase 6; PDLIM7, PDZ and LIM domain protein 7; SPINT2, serine protease inhibitor, Kunitz type 2; SVM, Support Vector Machine; TIMP3, tissue inhibitor of metalloproteinases 3; XGBoost, eXtreme Gradient Boosting.

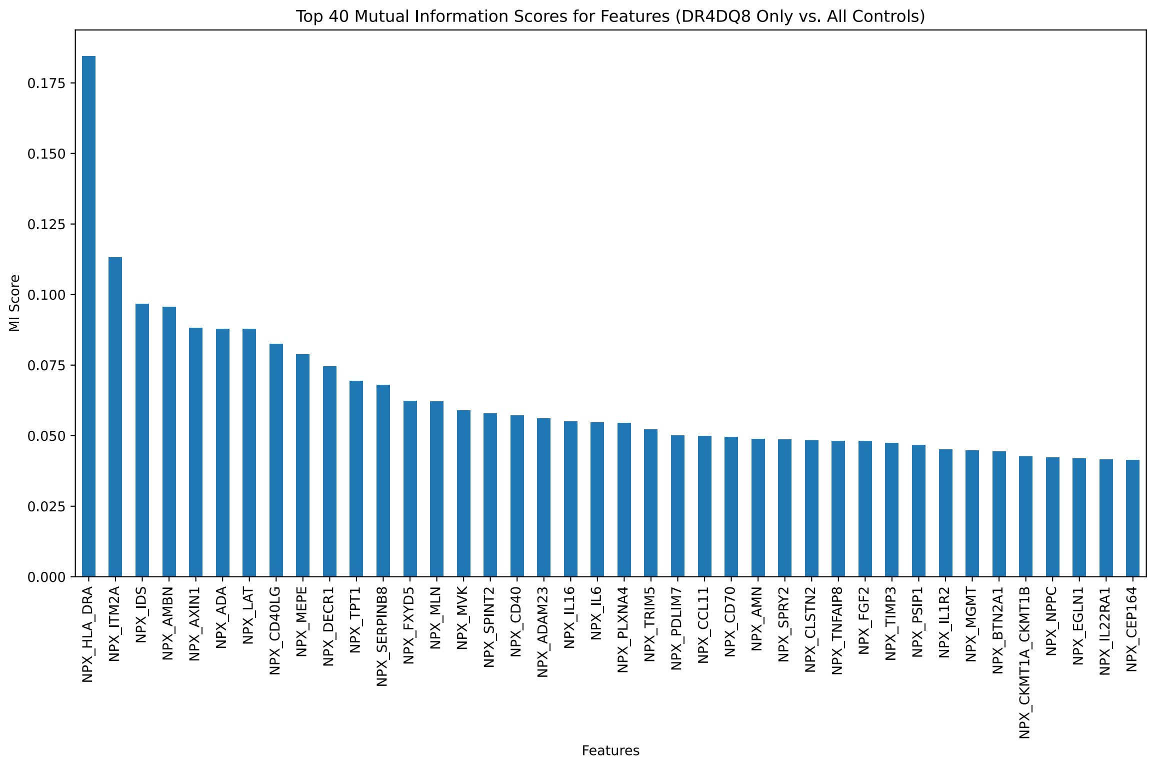

Supplementary Fig. 4. Significant proteins contributing to the machine learning model predicting type 1 diabetes (T1D) in cases carrying the HLA-DR4-DQ8 haplotype. Top 40 proteins selected as predictors for future T1D. The basis of this model was cases with the human leukocyte antigen (HLA) DR4-DQ8 allele (n=68), compared to all controls (irrespective of HLA genotype, n=268). Proteins are ranked by mutual information (MI) scores, reflecting their contribution to the machine learning model shown in Fig. 5f-g.

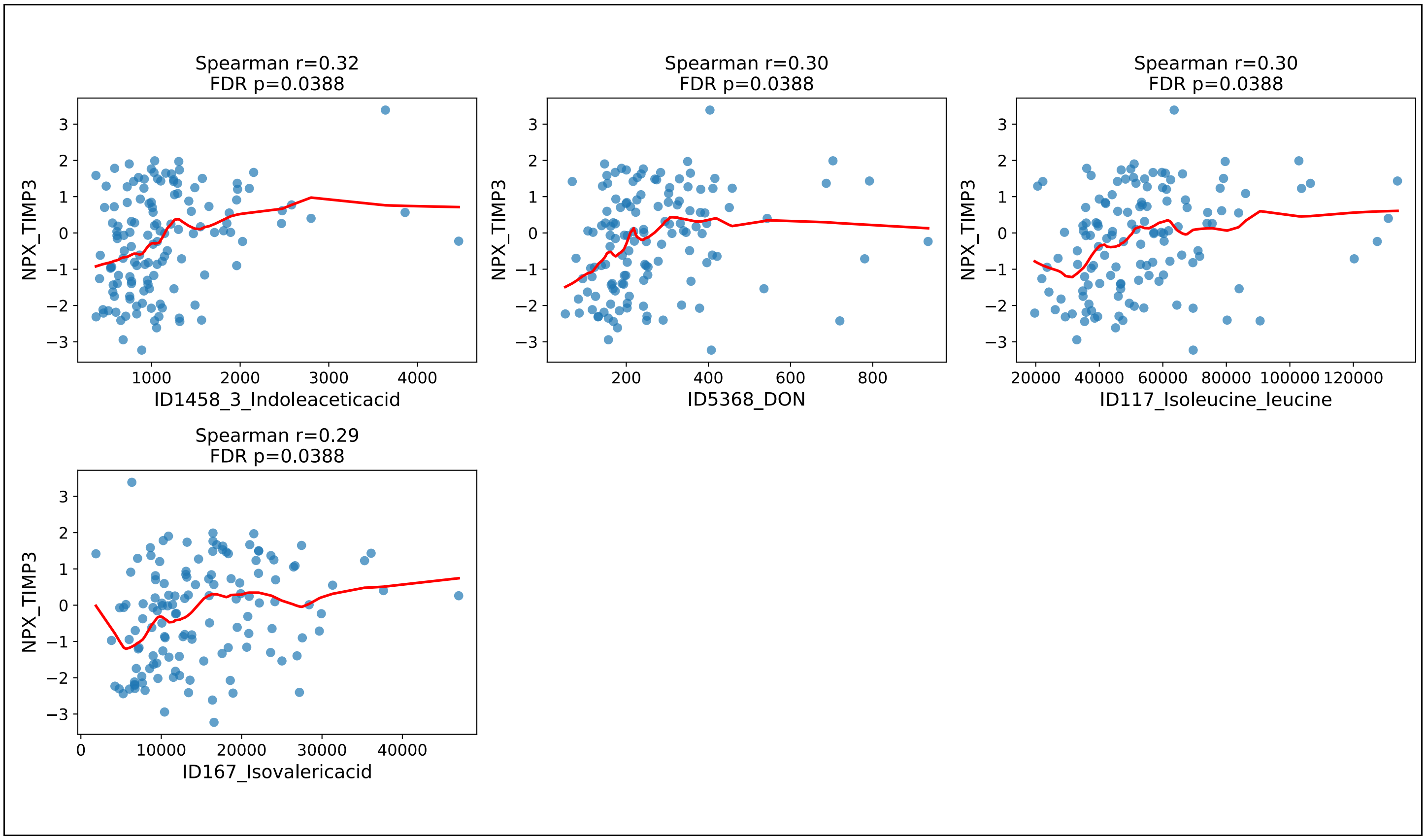

Supplementary Fig. 5. Non-parametric correlations of tissue inhibitor of metalloproteinases 3 (TIMP3) abundance with metabolite and toxin concentrations in the cord blood. Spearman correlations between protein levels and metabolites and exogenous compounds in the cord sera (n=132), significant after false discovery rate (FDR) correction. To counteract the influence of outlying subjects, the top 2.5% and bottom 2.5% of samples are removed. Correlations that are still significant after this step are presented for TIMP3, higher in controls compared to future type 1 diabetes (T1D). Variables with FDR-adjusted p-values < 0.05 are retained as significantly correlated. Abbreviations: DON, mycotoxin deoxynivalenol; NPX, normalized protein expression.
