# Supplementary material for: The inflammatory path toward type 1 diabetes begins during pregnancy
Source: Nat Commun. 2026 Jan 7;17:979. doi: 10.1038/s41467-025-67712-6 (PMC12847856; doi:10.1038/s41467-025-67712-6)
Supplement: Supplementary file 13 — Description of Additional Supplementary Files [file 41467_2025_67712_MOESM13_ESM.docx]

**Description of additional supplementary files**

**Supplementary Data 1**

Population characteristics in the ABIS cohort, grouped by future T1D status and controls. P-values indicate two-sided chi-square tests, with sample sizes listed for each variable. Only factors from the prenatal and birth questionnaires are included.

**Supplementary Data 2**

Pre- and perinatal characteristics in future T1D cases and controls. P-values for case-control comparisons were based on chi-square tests or means tests assuming unequal variances and are not corrected for multiple comparisons. All tests are two-sided.

**Supplementary Data 3**

Characteristic differences between ABIS participants included in the Olink analysis and the remainder of the cohort. Chi-square tests were used to compare birth characteristics, and all tests are two-sided.

**Supplementary Data 4**

Mean normalized protein expression (NPX) values for proteins measured by Olink assays, stratified by T1D group status.

**Supplementary Data 5**

Significant associations between protein levels and T1D status in the ABIS cohort. P-values were corrected for multiple comparisons, and both corrected and uncorrected values are provided. All tests are two-sided.

**Supplementary Data 6**

Descriptive statistics for NPX levels of proteins that were significant after FDR adjustment in the global case-control comparison (see Supplementary Table 4).

**Supplementary Data 7**

Gene set enrichment analysis (GSEA) results comparing cases and controls, showing enriched pathways and representative core enrichment proteins. All tests are two-sided.

**Supplementary Data 8**

Significant associations with future T1D, stratified by age at diagnosis. P-values were calculated using Wilcoxon non-parametric tests, with both original and FDR-corrected values provided. The age group tested relative to controls (0–5, 6–10, 11–17, 18+) is indicated. STRING identifiers and descriptions are provided for proteins significant after FDR correction in early-diagnosed cases (0–5 years). All tests are two-sided.

**Supplementary Data 9**

Functional enrichment of proteins differentially expressed in early-diagnosed T1D (by age 5). STRING analysis spans Biological Process, Molecular Function (GO), KEGG, Reactome, WikiPathways, Human Phenotype (Monarch), and UniProt keywords. Only proteins significant after FDR correction (see Supplementary Table 8) are included, with p-values corrected for multiple comparisons. All tests are two-sided.

**Supplementary Data 10**

Significant associations with future T1D, stratified by HLA genetic risk. P-values were calculated using Wilcoxon non-parametric tests, with both original and FDR-corrected values provided. The tested group relative to controls is indicated. All tests are two-sided.

**Supplementary Data 11**

Associations between prenatal and perinatal features and NPX protein expression. Wilcoxon tests were performed, with both original and FDR-corrected p-values provided. All tests are two-sided.
