# Supplementary material for: The inflammatory path toward type 1 diabetes begins during pregnancy
Source: Nat Commun. 2026 Jan 7;17:979. doi: 10.1038/s41467-025-67712-6 (PMC12847856; doi:10.1038/s41467-025-67712-6)
Supplement: Supplementary file 14 — Reporting Summary [file 41467_2025_67712_MOESM14_ESM.pdf]

nature portfolio

Corresponding author(s): Johnny Ludvigsson and Eric W. Triplett

Last updated by author(s): Nov 18, 2025

Reporting Summary

Nature Portfolio wishes to improve the reproducibility of the work that we publish. This form provides structure for consistency and transparency in reporting. For further information on Nature Portfolio policies, see our Editorial Policies and the Editorial Policy Checklist.

Statistics

For all statistical analyses, confirm that the following items are present in the figure legend, table legend, main text, or Methods section.

n/a	Confirmed	
☐	☒	The exact sample size (*n*) for each experimental group/condition, given as a discrete number and unit of measurement
☒	☐	A statement on whether measurements were taken from distinct samples or whether the same sample was measured repeatedly
☐	☒	The statistical test(s) used AND whether they are one- or two-sided<br>*Only common tests should be described solely by name; describe more complex techniques in the Methods section.*
☐	☒	A description of all covariates tested
☐	☒	A description of any assumptions or corrections, such as tests of normality and adjustment for multiple comparisons
☐	☒	A full description of the statistical parameters including central tendency (e.g. means) or other basic estimates (e.g. regression coefficient) AND variation (e.g. standard deviation) or associated estimates of uncertainty (e.g. confidence intervals)
☐	☒	For null hypothesis testing, the test statistic (e.g. *F*, *t*, *r*) with confidence intervals, effect sizes, degrees of freedom and *P* value noted<br>*Give P values as exact values whenever suitable.*
☒	☐	For Bayesian analysis, information on the choice of priors and Markov chain Monte Carlo settings
☒	☐	For hierarchical and complex designs, identification of the appropriate level for tests and full reporting of outcomes
☐	☒	Estimates of effect sizes (e.g. Cohen's *d*, Pearson's *r*), indicating how they were calculated

Our web collection on statistics for biologists contains articles on many of the points above.

Software and code

Policy information about availability of computer code

Data collection	No commercial software is used to collect the data for this study. All analyses are performed using open-source or freely available tools.<br>Primary comparative case–control analyses are carried out using the OlinkAnalyze R package (version 3.4.0). To adjust for potential confounding in T1D risk, median fold-change analyses are performed on a subset of controls matched 1:1 to cases using propensity score matching by nearest neighbor, implemented with the MatchIt R package (version 4.6.0).<br>Functional pathway enrichment analyses are carried out using the STRING database (version 12.0), restricted to proteins significantly associated with early-onset T1D (diagnosed by age five).<br>Machine-learning analyses—including XGBoost, Random Forest, Logistic Regression, and Support Vector Machines—are performed in Python within Jupyter Notebooks to ensure transparency and reproducibility. All model training procedures, hyperparameters, and evaluation methods are described in the main manuscript.
Data analysis	No custom software is used in this manuscript. Statistical packages in R and python are described.

For manuscripts utilizing custom algorithms or software that are central to the research but not yet described in published literature, software must be made available to editors and reviewers. We strongly encourage code deposition in a community repository (e.g. GitHub). See the Nature Portfolio guidelines for submitting code & software for further information.

Data

Policy information about availability of data

All manuscripts must include a data availability statement. This statement should provide the following information, where applicable:

- Accession codes, unique identifiers, or web links for publicly available datasets
- A description of any restrictions on data availability
- For clinical datasets or third party data, please ensure that the statement adheres to our policy

Provide your data availability statement here.

Research involving human participants, their data, or biological material

Policy information about studies with human participants or human data. See also policy information about sex, gender (identity/presentation), and sexual orientation and race, ethnicity and racism.

Reporting on sex and gender	Biological sex (male/female) is collected in the ABIS study from the birth questionnaire (reported by the parent) and is included in the study design. Gender identity is not collected; therefore, all analyses pertain solely to biological sex. The ABIS cohort shows a relatively balanced distribution of biological sex (48.2% female, 51.8% male), and the distribution of biological sex in the T1D case and control groups is broadly representative, but with a slight overrepresentation of females among controls included in the Olink proteomics subset (52.5%) compared with controls not included (47.9%).<br>We evaluate whether neonatal protein levels differ by biological sex. None of the significant proteins are among the markers associated with future T1D in our primary analyses.<br>Because gender identity is not measured and the study focuses on neonatal biological processes, no gender-based analyses are performed.
Reporting on race, ethnicity, or other socially relevant groupings	No socially constructed or socially relevant variables such as race, ethnicity, or socioeconomic status are collected in this study. The ABIS cohort consists primarily of children born in Sweden to Swedish parents, and although data on race, ethnicity, or ancestry are not gathered, the population is expected to be relatively homogeneous. At enrollment, 89.2% of ABIS children have both parents born in Sweden, 7.7% have one parent born outside Sweden, and 3.1% have both parents born outside Sweden. These data come from self-report, and serve only to contextualize the demographic structure of the cohort rather than classify participants into sociocultural groups.<br>Because these variables are not collected, we do not use race or ethnicity as proxies for any other social variable, and no analyses rely on such constructs. Potential confounding is instead addressed through biological and clinical variables available in the ABIS dataset (e.g., sex, gestational age, delivery mode, maternal diabetes, and serious life events), and these factors are evaluated in relation to the proteomic measurements. Associations between neonatal proteins and future T1D persist in spite of these corrections.
Population characteristics	Covariate-relevant population characteristics of the human participants are summarized in Supplementary Fig. 2. These include mode of delivery, sex, gestational age, family history of type 1 diabetes, maternal smoking during pregnancy, social vulnerability indicators, severe life events during pregnancy, maternal stomach flu during pregnancy, and parental education level.<br>All children in the study are born between October 1997 and 1999, and follow-up for type 1 diabetes diagnosis extends through December 2023. Case status is determined using ICD-10 code E10 from the Swedish National Patient Register. Controls have no documented psychological, neurodevelopmental, or autoimmune diagnoses through the end of follow-up. Proteomic measurements are generated from cord blood collected at birth.
Recruitment	Participants were recruited through the All Babies in Southeast Sweden (ABIS) cohort, which follows 16,683 children born between October 1997 and 1999 in southeastern Sweden. Families were approached at nine obstetric clinics across hospitals in the counties of Östergötland, Småland, Blekinge, and Öland and received both oral and written information before being invited to participate. Of the 21,700 children born in the region during the enrollment period, 78.6% of families provided informed consent. Cord blood samples were collected at birth from all enrolled children.<br>Some degree of self-selection bias may be present, as families who consent to participate may differ modestly from those who decline in terms of health awareness, engagement with healthcare systems, or sociodemographic characteristics. However, comparisons of key baseline characteristics between participants and the underlying birth population show broad representativeness of the enrolled cohort, suggesting that any potential selection bias is unlikely to meaningfully affect the main findings.
Ethics oversight	Ethical approval for the ABIS study is obtained from the Research Ethics Committees of the Faculty of Health Sciences at Linköping University (Ref. 1997/96287 and 2003/03-092) and the Medical Faculty of Lund University (Dnr 99227, Dnr 99321; prolongation of ABIS 03/092; adult follow-up 2019-05227). Linkage to national registers is approved under Dnr 03-513 and 2018/380-32. Multinational collaborations with the University of Florida are approved by the University of Florida Institutional Review Board (IRB201800903 and IRB202301239).

Note that full information on the approval of the study protocol must also be provided in the manuscript.

Field-specific reporting

Please select the one below that is the best fit for your research. If you are not sure, read the appropriate sections before making your selection.

☒ Life sciences ☐ Behavioural & social sciences ☐ Ecological, evolutionary & environmental sciences

For a reference copy of the document with all sections, see nature.com/documents/nr-reporting-summary-flat.pdf

Life sciences study design

All studies must disclose on these points even when the disclosure is negative.

Sample size	No formal a priori sample-size calculation was performed for this proteomics investigation. Instead, we included all available cord blood samples from ABIS participants who later developed type 1 diabetes (T1D) at the time of analysis, along with eligible controls. This approach maximizes statistical power in a rare-outcome cohort and reflects the full set of cases available. We have added text to the Methods section clarifying that sample size was determined by cohort availability and feasibility rather than predetermined calculations, and that the resulting sample size was sufficient to detect group-level differences across the proteome.
Data exclusions	Data exclusions are described in the main manuscript. For prenatal risk-factor analyses, questionnaire variables with ≥15% missingness were excluded according to pre-specified criteria. For the proteomic dataset, two samples failed Olink quality-control thresholds and were removed prior to analysis. For the primary case–control comparisons, controls were defined as participants without an E10 diagnosis and without any neurodevelopmental or autoimmune conditions through December 2023; cases were defined by the presence of an E10 diagnostic code. No other exclusions were made.
Replication	Reproducibility was evaluated using internal resampling-based approaches. For all machine-learning models, we used repeated cross-fold validation, as detailed in the Methods, to ensure stability of feature selection and predictive performance. The primary statistical findings (group differences and associations) were based on predefined analyses applied consistently across the cohort. No experimental findings required technical replication, and all analyses were computationally reproducible using the code and workflows described.
Randomization	Randomization was not applicable for participant allocation because this study used observational human cohort data. However, sample placement on proteomic assay plates was randomized to minimize potential batch effects. For machine-learning analyses, random seeds were set to ensure reproducibility during random subsampling procedures. Aside from these steps, randomization was not required because exposure and outcome status were determined solely by clinical follow-up and registry data, not assigned by investigators.
Blinding	Blinding was not relevant to this observational cohort study. Group status (future T1D vs. control) was determined independently through national registry follow-up using standardized diagnostic codes, and no experimental interventions were performed. All data processing and statistical analyses were conducted using objective computational methods, with no subjective scoring or investigator-dependent measurement. Because neither data collection nor analysis could be influenced by investigator knowledge of group allocation, blinding was not applicable.

Reporting for specific materials, systems and methods

We require information from authors about some types of materials, experimental systems and methods used in many studies. Here, indicate whether each material, system or method listed is relevant to your study. If you are not sure if a list item applies to your research, read the appropriate section before selecting a response.

Materials & experimental systems

n/a	Involved in the study	
☒	☐	Antibodies
☒	☐	Eukaryotic cell lines
☒	☐	Palaeontology and archaeology
☒	☐	Animals and other organisms
☐	☒	Clinical data
☒	☐	Dual use research of concern
☒	☐	Plants

Methods

n/a	Involved in the study	
☒	☐	ChIP-seq
☒	☐	Flow cytometry
☒	☐	MRI-based neuroimaging

Clinical data

Policy information about clinical studies

All manuscripts should comply with the ICMJE guidelines for publication of clinical research and a completed CONSORT checklist must be included with all submissions.

Clinical trial registration	Not applicable.
Study protocol	The full ABIS study protocol, as approved by ethical boards, is written in Swedish and is not publicly posted online. It is available upon

Study protocol	reasonable request from the ABIS principal investigator, Professor Johnny Ludvigsson (Linköping University). General information about the ABIS cohort and study procedures is available on the ABIS website. https://www.abis-studien.se/hem/english-11100423
Data collection	Participants were recruited through the All Babies in Southeast Sweden (ABIS) cohort, which follows 16,683 children born between October 1997 and 1999 in southeastern Sweden. Families were approached at nine obstetric clinics across hospitals in the counties of Östergötland, Småland, Blekinge, and Öland and received both oral and written information before being invited to participate. Of the 21,700 children born in the region during the enrollment period, 78.6% of families provided informed consent. Cord blood samples were collected at birth from all enrolled children. Follow-up is ongoing, to present day. Only the birth questionnaire was analyzed in this investigation, along with linkage to medical diagnoses from the Swedish National Patient Register.
Outcomes	Primary outcome is type 1 diabetes confirmed by a medical professional and is measured by E10 ICD-10 code in the Swedish National Patient Registry.

Plants

Seed stocks	*Report on the source of all seed stocks or other plant material used. If applicable, state the seed stock centre and catalogue number. If plant specimens were collected from the field, describe the collection location, date and sampling procedures.*
Novel plant genotypes	*Describe the methods by which all novel plant genotypes were produced. This includes those generated by transgenic approaches, gene editing, chemical/radiation-based mutagenesis and hybridization. For transgenic lines, describe the transformation method, the number of independent lines analyzed and the generation upon which experiments were performed. For gene-edited lines, describe the editor used, the endogenous sequence targeted for editing, the targeting guide RNA sequence (if applicable) and how the editor was applied.*
Authentication	*Describe any authentication procedures for each seed stock used or novel genotype generated. Describe any experiments used to assess the effect of a mutation and, where applicable, how potential secondary effects (e.g. second site T-DNA insertions, mosiacism, off-target gene editing) were examined.*
